# 

**Published:** 1884-12

**Authors:** 


					﻿I. H. A. Meeting for 1885.—The next meeting of the
International Hahnemann Association will be held at the Globe
Hotel, Syracuse, N. Y., Tuesday, June 23d, 1885, at 10 a. m.,
continuing three days. Geo. F. Foote, M. D.
Chairman Ex. Committee.
NOTICE.
All articles for publication, books for review, business communications, etc.,
must be sent to Editor, 129 South Thirteenth Street, Philadelphia.
				

